# The impact of COVID-19 lockdown on sleep patterns, emotions, and behaviors of children and adolescents in Taiwan

**DOI:** 10.3389/fpsyt.2022.975399

**Published:** 2022-08-22

**Authors:** Wei-Chih Chin, Tsung-Chieh Yao, I Tang, Pin-Yi Lee, Yu-Shu Huang

**Affiliations:** ^1^Division of Psychiatry and Sleep Center, Chang Gung Memorial Hospital, Taoyuan, Taiwan; ^2^College of Medicine, Chang Gung University, Taoyuan, Taiwan; ^3^Division of Allergy, Asthma, and Rheumatology, Department of Pediatrics, Chang Gung Memorial Hospital, Taoyuan, Taiwan; ^4^Department of Clinical Psychology, Fu Jen Catholic University, New Taipei City, Taiwan

**Keywords:** COVID-19, emotion, children, adolescents, sleep pattern, lockdown

## Abstract

**Background:**

The lockdown in May 2021 was the first and only lockdown implemented in Taiwan during the COVID-19 pandemic. The epidemic in Taiwan has been maintained under relatively better control and this study aimed to examine the impact of the lockdown on sleep and emotional and behavior disturbances in children and adolescents in Taiwan.

**Material and methods:**

Participants over 6 years old were recruited retrospectively from a cohort study, and their parents completed questionnaires including the Children's Sleep Habits Questionnaire (CSHQ), the Child Behavior Checklist (CBCL), the Swanson, Nolan and Pelham-IV Teacher and Parent Rating Scale (SNAP-IV), and the function assessment. A total of 217 children and adolescents whose parents completed questionnaires during both the lockdown period and the pre-lockdown period were included. We used paired *t*-test and independent *t* test; to analyze the differences between the lockdown and pre-lockdown periods and between different subgroups.

**Results:**

The mean age of all participants was 11.87 ± 3.97 years, with 69.6% male. The results of CSHQ indicated that our participants had significantly increased total sleep time (*p* = 0.000), more sleep onset delay (*p* = 0.011), fewer sleep duration problems (*p* = 0.029), less parasomnia (*p* = 0.018), fewer sleep breathing problems (*p* = 0.028), and less daytime sleepiness (*p* = 0.000) during the lockdown, especially males and children. We observed trends but no significant changes of all participants in CBCL and SNAP during the lockdown. The change of the inattention index reached a significant level between children and adolescents (*p*^2^ = 0.017). The results of the functional assessment showed more interferences in home living (*p* = 0.021) of all participants, especially males (*p*^1^ = 0.002).

**Conclusions:**

The lockdown significantly impacted children and adolescents' sleep and functioning. We also observed trends of increased emotion, behavior and inattention problems, and significantly increased interference in home living. Male gender and younger age may be associated factors for sleep and functional disturbances of the lockdown.

## Introduction

Since December 2019, the novel coronavirus disease (COVID-19) has rapidly spread around the world and become one of the major pandemics in human history (1). The World Health Organization (WHO) declared the COVID-19 outbreak a public health emergency of international concern on January 30, 2020 (2). Since then, the virus has spread all over the world. As of March 2022, COVID-19 has infected over 470 million people, and caused more than 6 million deaths (3). To contain the spread of infection, governments and local authorities have addressed the critical situation by implementing various measures. Examples include advocating the importance of mask wearing, quarantine, isolation, and social distancing. Other stricter measures included the mandatory closure of schools and the suspension of all nonessential production and commercial activities in order to minimize the social contact between people and clustering among people. Such measures can seriously affect people in multiple aspects, like daily life, work, and school activities (4).

While these restrictions can be necessary when fighting against a highly spreadable disease, the most extreme prevention “lockdown” has inevitably caused considerable impacts. Prolonged home confinement during the lockdown may have affected people's physical and mental health not only by increasing the level of stress related to social isolation (e.g., avoiding social contact with family and friends) (5) but also reducing the level of physical activity and exposure to daylight (6). Such circumstances can also lead to changes in the sense of time (7), disrupt daily lifestyle and night-time sleep (8), and result in other mental or behavioral health problems (9). A cross-sectional questionnaire-based survey of adults has demonstrated delayed bedtime and wake time during the lockdown period (10). Furthermore, at least one kind of sleeping difficulty was reported by adults during the home confinement without working, such as disturbed sleep quality, insomnia, or frequent awakenings (11).

Children and adolescents may experience higher rates of sleep issues and mood and behavioral problems during the lockdown (12–15). Substantial changes took place in the life of children and adolescents during the lockdown, from mandatory school attendance to online learning, interruption of in-person peer relationships, limited outdoor activity, and disruption of daily routines. With more flexible sleep and wake schedules and increased chances of longer nap times during the daytime, these changes can lead to unhealthy sleep patterns and sedentary lifestyles. In Italy, one of the early major hotspots of the COVID-19 pandemic, a study consisting of 5,805 participants found a significant delay in the bedtime and rise time of all ages (16), and the proportion of children and adolescents with a weekday bedtime after 11 p.m. remarkably increased to 27.5% in children and 51.2% in adolescents, compared to 0.9 and 12.3% before the pandemic. Children and adolescents also had increased emotional, conduct, and hyperactive symptoms, which could have been predicted by the change in sleep quality, boredom, and parents' psychological difficulties (12).

Taiwan is known for its excellent containment of the outbreak. However, the COVID-19 situation in Taiwan worsened on May 15, 2021, as shown by both cases of unknown sources of infection and cluster infections. The Central Epidemic Command Center (CECC) announced that in response to the severity of COVID-19 in Taiwan and an increasing level of community transmission, the epidemic warning level was raised to Level 3, and related national prevention measures were strengthened on May 15 (17) and extended nationwide on May 17, 2021. The heightened epidemic warning levels lasted more than 2 months, and during this period, children, adolescents, and their families faced the challenges of the sudden lockdown.

This lockdown represents the first and only lockdown in Taiwan so far, and referring to the lockdowns in other countries, it can impact the Taiwanese population, especially children, and adolescents. Exploring these impacts in Taiwan is of particular interest, since the epidemic in Taiwan was relatively better controlled until that point, and the goal of its epidemic control was still zero clearing. Furthermore, unlike the situations of previous studies (12, 16), vaccinations were available during the lockdown in Taiwan. Till now, there is no published study investigating psychosocial impacts of the lockdown in Taiwan. This study is a retrospective study from a 10-year cohort study of premature and mature infants and was carried out with the aim of examining the impact of the lockdown on sleep patterns and emotional and behavior disturbances in children and adolescents in Taiwan who were confined at home during the lockdown period. The results of this study may present the Taiwan experience of management of COVID and help to shed light on further cross-country comparisons.

## Materials and methods

### Participants: Inclusion and exclusion criteria

Participants over 6 years old (age range 6–18) were retrospectively recruited to investigate the impact of the COVID-19 pandemic lockdown on sleep, emotions, and behaviors in children and adolescents. 19.8% of our participants had ADHD, 12.0% had asthma history, 10.6% had tic history, and 4.6% had learning problems. They have received regular follow-up since the infant stage in the Pediatric Clinic of Neonatology and the Child Psychiatric Clinic of Chang Gung Memorial Hospital, Linkou Branch. Development, sleep patterns, emotions, behaviors, and learning were regularly evaluated, and questionnaires were completed every 6 months in the premature and mature cohort study. Therefore, we were able to compare the differences of our collected data from the period between June and August 2021 (the lockdown period, 3 months from 17 May 2021) and the period from October to December 2020 (the pre-lockdown period). Children and adolescents whose parents completed questionnaires during both the lockdown period and the pre-lockdown period were included, and those whose parents were unwilling to participate and did not complete questionnaires were excluded. This study was approved by the institutional review board of Chang Gung Memorial Hospital (CGMH #201801537A3 and 202200182B0). We divided participants into subgroups by gender and age, and the children group and the adolescent group were divided into over 12 years and under 12 years old.

### Measurements

All participants completed their scheduled visits during the two periods of time in 2020 and 2021. The following questionnaires were completed by participants' parents:

#### Children's sleep habits questionnaire (CSHQ)

CSHQ is a common questionnaires assessing children' sleep problem (18) and we used it to evaluate sleep changes during the lockdown. This is a 45-item multidimensional parent-reported measure that can be used to screen for the frequency of behaviors associated with common pediatric sleep difficulties in pre-school and school-aged children, including 33 scored questions and 7 additional items. Each question is rated from 1 to 3 points (1 = rarely, 2 = sometimes, 3 = usually) and some questions are reversely scored. It yields both a total score (range from 33 to 99) and eight subscale scores (bedtime resistance, sleep onset delay, sleep duration, sleep anxiety, night wakenings, parasomnia, sleep disordered breathing, and daytime sleepiness), reflecting key sleep domains that encompass the major medical and behavioral sleep disorders in children.

The internal consistency (alpha) of CSHQ was 0.68 and 0.78 for the community and clinical samples, respectively, and its test–retest reliability (interclass correlation) of the subscales ranged from 0.62 to 0.79 (18).

#### Child behavior checklist (CBCL)

CBCL evaluates children's emotions and behaviors and can be applied to children and adolescents 4–16 years old (19); it has a total of 137 items ranging from 0 (“not true”) to 2 (“very true”). The Chinese version of CBCL has been validated with good internal consistency (cronbach alpha = 0.81–0.93) and test retest reliability (intraclass correlation = 0.52–0.87) (20). The problem behavior items are loaded onto two broad-band scales (Internalizing and Externalizing) and eight narrow-band scales (Rule Breaking, Aggressive Behavior, Withdrawn-Depressed, Somatic Complaints, Anxious, Depressed, Social Problems, Thought Problems, and Attention Problems). The adaptive behavior items are loaded onto three scales: Activities, Social Competence, and School Competence (21). We used this checklist to evaluate changes in children's emotions and behaviors during the lockdown.

#### Swanson, nolan and pelham-IV teacher and parent rating scale (SNAP-IV)

We used the Chinese version of the Swanson, Nolan, and Pelham IV scale (SNAP-IV) (22), which has been validated by Gau et al. (22) and has similar three-factor structure as the English version to evaluate children's ADHD symptoms during the lockdown. The test-retest reliability, internal consistency and concurrent and discriminant validity are satisfactory (intraclass correlation = 0.59–0.72, alpha = 0.88–0.90, Pearson correlations = 0.56–0.72) (22). It is a widely used questionnaire that evaluates children and adolescents' inattention, hyperactivity/impulsivity, and oppositional/defiant symptoms. It consists of 26 items, including 18 items for attention deficit hyperactivity disorder (ADHD) symptoms (9 for inattention and 9 for hyperactivity/impulsivity) and 8 items for oppositional defiant disorder (ODD) symptoms. All items are rated on a 4-point scale from 0 to 3 (0 = not at all, 1 just a little, 2 quite a bit, and 3 very much), and higher scores indicate more severe symptoms. The Inattention Index, Hyperactivity Index and ODD index were calculated by the number of items which are scored 2–3. The Inattention total, Hyperactivity total and ODD total were calculated by the sum of scores.

#### The functional assessment

In the clinical evaluation of children's and adolescent's function, we referred to the evaluation of function disturbance in the Attention-Deficit Hyperactivity Disorder: A Clinical Workbook, Third Edition (23). We interviewed parents to assess the function disturbance in eight different situations, including home living, peer interaction, community activities, school studying, school clubs, self-care, leisure activities, duties, and assignments. Parents rated the disturbance as never/seldom, sometimes, often, and always (score 0–3). Since several situations were not applicable during the lockdown, only the results of the following four situations: home living, self-care, leisure activities, and duties and assignments, were collected in this study.

### Statistical analyses

We used SPSS 22.0 to analyze our data. Demographic data were presented as number, mean, percentage, and standard deviation. We used paired *t*-tests to analyze the differences between the data from 2020 and 2021, and independent *t* test to compare the changes between the pre-lockdown and lockdown periods of different subgroups (gender and age groups). A *p*-value of <0.05 was considered statistically significant.

## Results

We included a total of 217 participants in this study. The mean age of all participants was 11.87 ± 3.97 years, and 151 were male (69.6%). The children group (<12 years old) had 136 participants, with 64.7% male, and the adolescents group (more than 12 years old) had 81 participants, with 77.8% male.

### Comparison of CSHQ during the lockdown and pre-lockdown periods

Table 1A showed the differences of CSHQ between the lockdown and the pre-lockdown periods. The results of CSHQ demonstrated that our participants had significantly increased total sleep time (*p* = 0.000), more sleep onset delay (*p* = 0.011), fewer sleep duration problems (*p* = 0.029), less parasomnia (*p* = 0.018), fewer sleep breathing problems (*p* = 0.028), and less daytime sleepiness (*p* = 0.000) during the lockdown, as well as a trend of increased bedtime resistance (*p* = 0.062). Sleep disorder breathing was not significantly different between the pre-lockdown and lockdown periods if the result was analyzed by the Wilcoxon signed-rank test (*p* = 0.051). Table 1B showed the comparison of CSHQ of different genders between the lockdown and pre-lockdown periods. We found that males had significantly increased total sleep time (*p*1 = 0.000), more sleep onset delay (*p*1 = 0.006), less parasomnia (*p*1 = 0.028), and less daytime sleepiness (*p*1 = 0.002) during the lockdown period. Although it was not statistically significant, females had a trend of decreased sleep duration problems (*p*1 = 0.05) during the lockdown period. Beside, total sleep time of females was significantly increased during the lockdown period if the result was analyzed by the Wilcoxon signed-rank test (*p* = 0.022). We found no significant difference in CSHQ changes between genders during the lockdown and pre-lockdown periods. Table 1C shows the comparison of CSHQ of different age groups (the children group, which is <12 years old, and the adolescents group, which is more than 12 years old) between the lockdown and pre-lockdown periods. The children group had significantly increased total sleep time (*p*1 = 0.000), more sleep onset delay (*p*1 = 0.019), fewer sleep duration problems (*p*1 = 0.046), fewer sleep breathing problems (*p*1 = 0.009), and less daytime sleepiness (*p*1 = 0.001) during the lockdown period. The adolescents group also had significantly increased total sleep time (*p*1 = 0.008), and the increase was significantly more than the children group (*p*2 = 0.012).

**Table 1A:** Comparison of CSHQ during the lockdown and pre-lockdown periods.

**CSHQ variables**	**pre-test (pre-lockdown)**	**post-test (lockdown)**	***p* value**
	**M±SD (N = 217)**	**M±SD (N = 217)**	
Sleep time (pm)	22:04	22:23	
Awake time (am)	6:56	7:47	
Total sleep time (minutes)	533.52 ± 70.16	573.30 ± 65.41*	0.000*
Bedtime resistance	8.65 ± 2.62	9.02 ± 2.98	0.062
Sleep onset delay	1.50 ± 0.71	1.66 ± 0.77	0.011*
Sleep anxiety	6.06 ± 2.12	6.14 ± 2.26	0.638
Sleep duration	4.90 ± 1.62	4.57 ± 1.42	0.029*
Parasomnias	8.93 ± 1.85	8.58 ± 1.78	0.018*
Night wakenings	3.83 ± 1.39	3.90 ± 1.36	0.538
Sleep disordered breathing	3.98 ± 1.30	3.75 ± 1.19	0.028*
Daytime sleepiness	14.08 ± 3.31	12.82 ± 3.16	0.000*

M+SD, mean ± standard deviation; paired t-test; *p < 0.05. CSHQ, Children's Sleep Habits Questionnaire, higher scores indicate more sleep problems, except for Sleep Duration; a higher score in Sleep Duration indicates less of a sleep duration problem.

**Table 1B:** Comparison of CSHQ of different genders during the lockdown and pre-lockdown periods.

**CSHQ variables**	**Male (*****n*** = **151)**			**Female (*****n*** = **66)**			***p2***
	**pre-test**	**post-test**	***p1***	**pre-test**	**post-test**	***p1***	
	**(pre-lockdown)**	**(lockdown)**		**(pre-lockdown)**	**(lockdown)**		
Sleep time (pm)	22:08	22:28		21:54	22:13		
Awake time (am)	6:58	7:52		6:48	7:34		
Total sleep time (minutes)	532.17 ± 66.25	577.73 ± 60.47	0.000*	537.14 ± 80.91	561.43 ± 77.07	0.130	0.233
Bedtime resistance	8.71 ± 2.64	8.99 ± 2.98	0.200	8.50 ± 2.61	9.11 ± 3.02	0.174	0.449
Sleep onset delay	1.43 ± 0.65	1.61 ± 0.76	0.006*	1.69 ± 0.82	1.78 ± 0.80	0.539	0.470
Sleep anxiety	6.04 ± 2.06	6.09 ± 2.25	0.804	6.11 ± 2.31	6.26 ± 2.31	0.632	0.773
Sleep duration	4.97 ± 1.56	4.73 ± 1.39	0.192	4.75 ± 1.76	4.17 ± 1.44	0.051	0.304
Parasomnias	8.93 ± 1.79	8.57 ± 1.77	0.028*	8.91 ± 2.05	8.60 ± 1.82	0.324	0.874
Night wakenings	3.87 ± 1.40	3.95 ± 1.49	0.569	3.71 ± 1.36	3.77 ± 0.91	0.804	0.944
Sleep disordered breathing	4.05 ± 1.35	3.80 ± 1.24	0.054	3.77 ± 1.17	3.63 ± 1.09	0.257	0.527
Daytime sleepiness	14.05 ± 3.37	12.70 ± 3.25	0.002*	14.18 ± 3.18	13.15 ± 2.93	0.066	0.680

Results are expressed as mean ± standard deviation; p1, paired t-test; p2, independent t test; *p < 0.05.

CSHQ, Children's Sleep Habits Questionnaire, higher scores indicate more sleep problems, except for Sleep Duration; a higher score in Sleep Duration indicates less of a sleep duration problem.

**Table 1C:** Comparison of CSHQ of different age groups during the lockdown and pre-lockdown periods.

**CSHQ variables**	**Under 12 years old (N** = **136)**			**Above 12 years old (N** = **81)**			***p2***
	**pre-test**	**post-test**	***p1***	**pre-test**	**post-test**	***p1***	
	**(pre-lockdown)**	**(lockdown)**		**(pre-lockdown)**	**(lockdown)**		
Sleep time (pm)	21:50	21:58		22:28	22:52		
Awake time (am)	6:55	7:39		6:57	8:01		
Total sleep time (minutes)	545.7 ± 58.75	576 ± 58.92	0.000*	478.63 ± 47.7	568.18 ± 89.53	0.008*	0.012*
Bedtime resistance	9.00 ± 2.69	9.32 ± 3.05	0.151	7.00 ± 1.38	7.64 ± 2.19	0.188	0.543
Sleep onset delay	1.53 ± 0.71	1.69 ± 0.80	0.019*	1.36 ± 0.66	1.50 ± 0.60	0.329	0.901
Sleep anxiety	6.31 ± 2.17	6.38 ± 2.27	0.697	4.91 ± 1.41	5.00 ± 1.88	0.765	0.955
Sleep duration	4.74 ± 1.57	4.40 ± 1.33	0.046*	5.71 ± 1.65	5.38 ± 1.60	0.398	0.994
Parasomnias	8.99 ± 1.90	8.69 ± 1.80	0.063	8.60 ± 1.57	8.00 ± 1.52	0.104	0.463
Night wakenings	3.91 ± 1.47	3.93 ± 1.33	0.821	3.45 ± 0.80	3.73 ± 1.49	0.342	0.420
Sleep disordered breathing	3.93 ± 1.24	3.65 ± 1.10	0.009*	4.18 ± 1.56	4.23 ± 1.51	0.877	0.225
Daytime sleepiness	14.01 ± 3.43*	12.80 ± 3.17	0.001*	14.43 ± 2.71	12.95 ± 3.19	0.146	0.773

Results are expressed as mean ± standard deviation; p1, paired t-test; p2, independent t test; *p < 0.05.

CSHQ, Children's Sleep Habits Questionnaire, higher scores indicate more sleep problems, except for Sleep Duration; a higher score in Sleep Duration indicates less of a sleep duration problem.

### Comparison of CBCL during the lockdown and pre-lockdown periods

Table 2A shows that the results of CBCL did not differ significantly during the lockdown period, but we observed non-significant trends of increased internalizing problems, depression, and aggression during the lockdown period (more emotional and behavioral disturbance). Table 2B shows the comparison of CBCL of different genders between the lockdown and pre-lockdown periods. No significant difference was found, but we did find non-significant trends of increased internalizing problems, depression, aggression, and conduct problems in males during the lockdown period. Table 2C shows the comparison of CBCL of different age groups between the lockdown and pre-lockdown periods. Although no significant difference was observed, the adolescents group also had higher scores in internalizing and externalizing problems, obsession/compulsion, somatic complaints, and aggression during the lockdown.

**Table 2A:** Comparison of CBCL during the lockdown and pre-lockdown periods.

**CBCL variables**	**pre-test (pre-lockdown)**	**post-test (lockdown)**	***p* value**
	**M±SD (N = 217)**	**M±SD (N = 217)**	
Internalizing problems	53.40 ± 11.76	53.64 ± 10.73	0.788
Externalizing problems	56.53 ± 11.35	56.18 ± 12.70	0.741
Anxiety	52.52 ± 9.35	51.53 ± 9.15	0.187
Depression	55.73 ± 9.11	56.24 ± 10.30	0.564
Obsession/compulsion	55.39 ± 9.84	54.74 ± 9.93	0.459
Somatic complaints	50.66 ± 8.00	50.32 ± 8.81	0.678
Social withdraw	55.06 ± 9.98	54.03 ± 11.58	0.324
Attention problems	59.33 ± 11.53	58.93 ± 11.19	0.604
Aggressive behavior	55.31 ± 12.24	56.55 ± 10.48	0.236
Conduct problems	56.39 ± 9.46	55.78 ± 11.36	0.481

M+SD, mean ± standard deviation; paired t-test; *p < 0.05.

CBCL, Child Behavior Checklist, lower scores indicate fewer emotion and behavior problems.

**Table 2B:** Comparison of CBCL of different genders during the lockdown and pre-lockdown periods.

**CBCL variables**	**Male (*****n*** = **151)**			**Female (*****n*** = **66)**			***p2***
	**pre-test**	**post-test**	***p1***	**pre-test**	**post-test**	***p1***	
	**(pre-lockdown)**	**(lockdown)**		**(pre-lockdown)**	**(lockdown)**		
Internalizing problems	52.86 ± 11.99	53.72 ± 10.45	0.415	54.73 ± 11.26	53.43 ± 11.57	0.387	0.256
Externalizing problems	57.62 ± 9.60	56.95 ± 12.66	0.621	53.94 ± 14.58	54.35 ± 12.81	0.791	0.640
Anxiety	50.71 ± 8.72	50.62 ± 8.69	0.903	56.77 ± 9.53	53.68 ± 9.98	0.071	0.065
Depression	55.19 ± 8.83	56.12 ± 10.48	0.388	56.80 ± 9.70	56.47 ± 10.09	0.828	0.496
Obsession/compulsion	54.96 ± 10.36	54.82 ± 9.96	0.900	56.47 ± 8.49	54.53 ± 10.04	0.215	0.357
Somatic complaints	50.35 ± 7.02	50.24 ± 9.15	0.910	51.43 ± 10.13	50.50 ± 8.05	0.583	0.655
Social withdraw	55.07 ± 10.37	53.96 ± 12.13	0.391	55.03 ± 9.12	54.21 ± 10.29	0.635	0.902
Attention problems	59.38 ± 10.88	59.03 ± 1.07	0.689	59.23 ± 13.17	58.71 ± 11.65	0.747	0.923
Aggressive behavior	56.45 ± 10.85	57.54 ± 11.02	0.366	52.58 ± 14.89	54.19 ± 8.78	0.747	0.823
Conduct problems	56.27 ± 8.44	56.28 ± 12.18	0.990	56.67 ± 11.78	54.53 ± 9.06	0.107	0.258

Results are expressed as mean ± standard deviation; p1, paired t-test; p2, independent t test; *p < 0.05.

CBCL, Child Behavior Checklist, lower scores indicate fewer emotion and behavior problems.

**Table 2C:** Comparison of CBCL of different age groups during the lockdown and pre-lockdown periods.

**CBCL variables**	**Under 12 years old (N** = **136)**			**Above 12 years old (N** = **81)**			***p2***
	**pre-test**	**post-test**	***p1***	**pre-test**	**post-test**	***p1***	
	**(pre-lockdown)**	**(lockdown)**		**(pre-lockdown)**	**(lockdown)**		
Internalizing problems	53.82 ± 11.57	53.57 ± 10.69	0.804	51.29 ± 12.82	53.94 ± 11.29	0.113	0.213
Externalizing problems	56.94 ± 11.55	56.45 ± 13.09	0.698	54.41 ± 10.34	54.76 ± 10.67	0.731	0.602
Anxiety	52.42 ± 8.77	51.53 ± 9.18	0.253	53.06 ± 12.42	51.50 ± 9.29	0.529	0.745
Depression	55.61 ± 9.07	56.24 ± 10.33	0.496	58.25 ± 11.24	56.25 ± 11.06	0.182	0.537
Obsession/compulsion	56.15 ± 9.62	54.83 ± 9.44	0.187	51.53 ± 10.35	54.29 ± 12.51	0.096	0.086
Somatic complaints	50.18 ± 7.49	49.46 ± 8.55	0.441	53.12 ± 10.16	54.71 ± 9.08	0.363	0.306
Social withdraw	54.90 ± 9.86	53.71 ± 11.96	0.323	56.00 ± 10.95	55.87 ± 9.20	0.929	0.582
Attention problems	59.41 ± 11.91	58.95 ± 11.76	0.597	58.94 ± 9.68	58.82 ± 7.84	0.948	0.873
Aggressive behavior	55.53 ± 12.56	56.91 ± 10.24	0.261	54.12 ± 10.68	54.71 ± 11.82	0.710	0.783
Conduct problems	56.37 ± 9.62	55.91 ± 11.62	0.638	56.47 ± 8.89	55.12 ± 10.20	0.422	0.702

Results are expressed as mean ± standard deviation; p1, paired t-test; p2, independent t test; *p < 0.05.

CBCL, Child Behavior Checklist, lower scores indicate fewer emotion and behavior problems.

### Comparison of SNAP during the lockdown and pre-lockdown periods

Table 3A shows the results of SNAP and the functional assessment. Although any differences in SNAP were not significant, most parameters were scored higher during the lockdown. The functional assessment showed significantly more interference in home living (*p* = 0.021). Table 3B shows the comparison of SNAP and the functional assessment of different gender groups between the lockdown and pre-lockdown periods. Males had higher but non-significant scores in all parameters of SNAP during the lockdown period, and more function disturbance in home living (*p*1 = 0.002). The changes of the function disturbance in home living reached significance between male and female (*p*2 = 0.005). Table 3C shows the comparison of SNAP and the functional assessment of different age groups between the lockdown and pre-lockdown periods, indicating no significant difference in SNAP or function assessment. The children group had non-significant increased inattention index, and the change of the inattention index reached significance between the children and adolescent groups (*p*2 = 0.017). The children group also had non-significantly more disturbance in home living (*p*1 = 0.054) and duties and assignments (*p*1 = 0.062) of the functional assessment during the lockdown period, but the disturbance in home living was significantly different if the result was analyzed by the Wilcoxon signed-rank test (*p* = 0.048).

**Table 3A:** Comparison of SNAP and the functional assessment during the lockdown and pre-lockdown periods.

**SNAP-IV variables**	**pre-test (pre-lockdown)**	**post-test (lockdown)**	***p* value**
	**M±SD (N = 217)**	**M±SD (N = 217)**	
Inattention index	2.81 ± 2.76	2.94 ± 2.73	0.546
Inattention total	10.19 ± 5.85	10.34 ± 5.64	0.719
Hyperactivity index	1.80 ± 2.36	1.84 ± 2.29	0.822
Hyperactivity total	7.17 ± 5.64	7.11 ± 5.62	0.863
ADHD total	17.36 ± 10.63	17.45 ± 10.28	0.896
ODD index	1.47 ± 2.26	1.59 ± 2.21	0.564
ODD total	6.16 ± 5.33	6.26 ± 5.31	0.823
**Functional assessment**
Life1 home living	0.81 ± 0.77	0.99 ± 0.86	0.021*
Life6 self-care	0.81 ± 0.79	0.79 ± 0.79	0.791
Life7 leisure activities	0.47 ± 0.66	0.42 ± 0.64	0.487
Life8 duties and assignments	0.90 ± 0.81	1.02 ± 0.83	0.186

M+SD, mean ± standard deviation; paired t-test; *p < 0.05. ADHD, attention deficit/hyperactivity disorder; ODD, oppositional defiant disorder; SNAP-IV, Swanson; Nolan and Pelham-IV Teacher and Parent Rating Scale, higher scores indicate more severe symptoms. The function assessment: higher scores indicate more disturbance.

**Table 3B:** Comparison of SNAP and the functional assessment of different genders during the lockdown and pre-lockdown periods.

**SNAP-IV variables**	**Male (*****n*** = **151)**			**Female (*****n*** = **66)**			***p2***
	**pre-test**	**post-test**	**p1**	**pre-test**	**post-test**	**p1**	
	**(pre-lockdown)**	**(lockdown)**		**(pre-lockdown)**	**(lockdown)**		
Inattention index	3.06 ± 2.70	3.22 ± 2.69	0.559	2.14 ± 2.83	2.22 ± 2.73	0.847	0.883
Inattention total	11.09 ± 5.20	11.23 ± 5.37	0.764	7.84 ± 6.82	8.00 ± 5.73	0.844	0.975
Hyperactivity index	2.07 ± 2.31	2.21 ± 2.40	0.512	1.08 ± 2.37	0.86 ± 1.65	0.431	0.345
Hyperactivity total	8.10 ± 5.18	8.20 ± 5.64	0.816	4.73 ± 6.13	4.27 ± 4.49	0.515	0.477
ADHD total	19.20 ± 9.30	19.42 ± 10.04	0.765	12.54 ± 12.40	12.27 ± 9.17	0.857	0.746
ODD index	1.69 ± 2.26	1.93 ± 2.33	0.398	0.90 ± 2.18	0.72 ± 1.58	0.510	0.284
ODD total	6.62 ± 5.08	7.03 ± 5.48	0.501	4.97 ± 5.85	4.31 ± 4.37	0.372	0.319
**Functional assessment**
Life1 home living	0.84 ± 0.64	1.15 ± 0.81	0.002*	0.72 ± 1.03	0.59 ± 0.87	0.255	0.005*
Life6 self-care	0.89 ± 0.80	0.89 ± 0.80	1.000	0.59 ± 0.73	0.52 ± 0.69	0.537	0.673
Life7 leisure activities	0.51 ± 0.63	0.47 ± 0.67	0.605	0.38 ± 0.73	0.31 ± 0.54	0.646	0.859
Life8 duties and assignments	1.03 ± 0.78	1.16 ± 0.78	0.199	0.59 ± 0.82	0.66 ± 0.86	0.677	0.736

Results are expressed as mean ± standard deviation; p1, paired t-test; p2, independent t test; *p < 0.05. ADHD, attention deficit/hyperactivity disorder; ODD, oppositional defiant disorder; SNAP-IV, Swanson; Nolan and Pelham-IV Teacher and Parent Rating Scale, higher scores indicate more severe symptoms. The function assessment: higher scores indicate more disturbance.

**Table 3C:** Comparison of SNAP and the functional assessment of different age groups during the lockdown and pre-lockdown periods.

**SNAP-IV variables**	**Under 12 years old (N** = **136)**			**Above 12 years old (N** = **81)**			***p2***
	**pre-test**	**post-test**	**p1**	**pre-test**	**post-test**	**p1**	
	**(pre-lockdown)**	**(lockdown)**		**(pre-lockdown)**	**(lockdown)**		
Inattention index	2.72 ± 2.77	3.10 ± 2.83	0.113	3.21 ± 2.73	2.21 ± 2.08	0.073	0.017*
Inattention total	10.05 ± 5.92	10.55 ± 5.79	0.249	10.88 ± 5.59	9.38 ± 4.90	0.106	0.050
Hyperactivity index	1.95 ± 2.45	2.05 ± 2.36	0.641	1.08 ± 1.74	0.88 ± 1.65	0.396	0.337
Hyperactivity total	7.58 ± 5.77	7.65 ± 5.72	0.858	5.29 ± 4.66	4.63 ± 4.43	0.221	0.414
ADHD total	17.62 ± 10.91	18.20 ± 10.53	0.455	16.17 ± 9.35	14.00 ± 8.40	0.112	0.123
ODD index	1.51 ± 2.31	1.61 ± 2.16	0.669	1.25 ± 1.98	1.50 ± 2.50	0.638	0.809
ODD total	6.30 ± 5.27	6.64 ± 5.19	0.513	5.38 ± 5.78	4.19 ± 5.65	0.304	0.246
**Functional assessment**
Life1 home living	0.86 ± 0.80	1.03 ± 0.90	0.054	0.53 ± 0.51	0.76 ± 0.56	0.163	0.776
Life6 self-care	0.83 ± 0.80	0.86 ± 0.78	0.664	0.71 ± 0.77	0.41 ± 0.71	0.096	0.094
Life7 leisure activities	0.49 ± 0.68	0.45 ± 0.61	0.550	0.35 ± 0.49	0.29 ± 0.77	0.718	0.951
Life8 duties and assignments	0.87 ± 0.82	1.05 ± 0.87	0.062	1.06 ± 0.75	0.88 ± 0.60	0.484	0.138

Results are expressed as mean ± standard deviation; p1, paired t-test; p2, independent t test; *p < 0.05. ADHD, attention deficit/hyperactivity disorder; ODD, oppositional defiant disorder; SNAP-IV, Swanson; Nolan and Pelham-IV Teacher and Parent Rating Scale, higher scores indicate more severe symptoms.

The function assessment: higher scores indicate more disturbance.

## Discussion

This study is the first to investigate children and adolescents' emotions, behaviors, and sleep during the lockdown in Taiwan. This study can increase understanding of the influence of the lockdown on children and adolescents, as well as provide comparisons between different countries with different pandemic severity and different disease control strategies.

Parents observed substantial changes in children and adolescents' sleep during the lockdown. We found that children and adolescents slept 40 min more and had fewer sleep duration problems, less parasomnia, fewer sleep breathing problems, and less daytime sleepiness, along with a trend of decreased bedtime resistance, but they also had more delayed sleep onset. Other studies also found that children were sleeping longer and later during the pandemic (24–28) and lockdown (16, 29, 30), that is, the pandemic has had both positive and negative impacts on sleep. Similar to our results, some studies have found that adolescents had less daytime sleepiness during the pandemic and school shutdown (24, 31). Sleep insufficiency in children and adolescents has been long observed in modern society, which has been related to academic stress and other psychosocial factors, such as school settings (32). The lockdown might provide an opportunity for children and adolescents to sleep more due to the school shutdown and study from home. Although the lockdown can disturb their daily living, adequate sleep and less academic stress may decrease such sleep disturbances as parasomnia and improve daytime sleepiness. However, with a less structured home setting than the school setting, decreased physical activity (33, 34), and increased screen time related to online studying and gaming (35, 36), they may also have sleep onset delay and shorter sleep duration.

The sleep analysis of the subgroups showed no significant change in the female group. Only males had significantly more total sleep time, less parasomnia, less daytime sleepiness, and more sleep onset delay. Although the total sleep time of both children and adolescents increased during the lockdown, children can be influenced more than adolescents. Only children had more sleep onset delay, fewer sleep duration problems, fewer sleep breathing problems, and less daytime sleepiness, and the increase of total sleep time of children was less than that of adolescents. Previous studies also found that sleep problems differ with age and gender (37–39), and most studies investigating youth sleep reported more sleep problems in adolescence and the female gender, such as delayed circadian rhythm disorder (40) and insomnia (41). Although female gender and adolescence have been identified as risk factors of sleep problems in youth, our results suggest that younger age and male gender can be associated factors for sleep problems related to the lockdown. Boys are more active than girls, and physical activity declines rapidly from childhood to adolescence (42); as a result, home confinement can have a greater impact on children and the male gender. Bruni et al. (16) also found that during home confinement, children had more sleep changes, including increased difficulty and anxiety at bedtime, night awakenings, nightmares and sleep terrors, but adolescents did not have the same experience, since the physiological sleep of adolescents was more delayed and fit the sleep pattern during the lockdown.

Although our results showed no significant increase in emotional or behavioral problems, we observed trends of increased internalizing problems, depression, and aggression during the lockdown. We found gender to play a role in the emotion/behavior problems and function disturbance related to the lockdown as only males showed trends of increased emotional and behavioral problems. Subgroup analysis also showed that adolescents had non-significantly more emotion and behavior problems than children. As previously mentioned, male gender may have been influenced more by the lockdown. Although gender differences in oppositional behavior is not certain, conduct problems such as aggression can be more common among boys (43, 44). Furthermore, gender difference in psychosocial adjustment was found particularly in terms of prosocial behavior in girls and behavioral problems in boys (45). Some studies have reported that female gender can correlate with anxiety and depression (46, 47), while others don't find any gender differences (48, 49). Age difference has also been investigated and similar to our finding, Liu et al. (48) has founds adolescents can have more anxiety, depression and somatization than school aged children, and another study has reported more depression and depression while age increases (46). Adolescents may have more emotional and behavior problems in line with the clinically reported increase in parent-adolescent conflicts. Previous studies have examined links between adolescents' peer relations and mental health (50, 51), and peer interactions interrupted by the lockdown may have contributed to the increase in emotion and behavior problems. However, there were several studies reporting no age difference of the impact of the pandemic (47–49, 52). Overall, current literatures of gender and age difference of the impact of COVID-19 in young people didn't have consistent results.

Though non-significant, the results of SNAP revealed trends of increased inattention, hyperactivity, and oppositional behavior during the lockdown. Subgroup analysis showed that males and children could be more vulnerable, and the difference between genders reached significance in the inattention index. Parents also reported function disturbance in the home living category. Males had more disturbance in home living, which reached significance between male and female, and children demonstrated trends of more function disturbance in both home living and duty and assignments. Complying with home rules set by parents and doing assignments through online teaching can be challenging. Children usually need more supervision in such situations as home living and assignments due to their development stage. Although most participants do not have attention deficit/hyperactivity disorder, children and males have a higher prevalence of the disorder and trait (53, 54). A more structured learning schedule and daily routine, as well as parenting discussion, should be provided to assist with their adjustment.

A wide range of negative consequences were reported since the outbreak of COVID-19 for children and adolescents, including an increase in sleep disturbances, depressive, and anxiety symptoms, and suicidal ideation and even potential delays in language and motor developments (55, 56). However, most present studies were cross-sectional and conducted online, and the changes may be transitory, being most prevalent at the beginning of the pandemic (57, 58). Compared with other studies, although we observed trends of increase in emotion and behavior problems, changes of CBCL and SNAP did not reach statistical significance. Our results showed that children and adolescents in Taiwan had fewer negative influences from the lockdown, especially regarding emotion and behavior. Possible reasons may include the relatively smaller sample size. Besides, personal hygiene such as mask wearing is widely accepted and the health insurance system is very accessible and cost effective in Taiwan. Furthermore, academic stress may be decreased (31), while the stress directly related to the lockdown may be relatively less in Taiwan due to better pandemic control. Palmer and Oosterhoff (59) found a bidirectional association between adolescents' sleep and mental health during the COVID-19 pandemic. With better pandemic control, less stress and fewer mental health problems may result in fewer sleep disturbances in children and adolescents, and vice versa. Negative changes in the work situation of parents due to COVID-19 regulations or a relative/friend infected with COVID-19 have also been identified as risk factors of worse mental health in children and adolescents (55), and these factors can be less common if the severity of the pandemic does not escalate.

This study has several limitations. First, the study design was retrospective, and no questionnaire developed specifically for the pandemic is currently available. Second, only subjective measurements were used, and the questionnaires were filled out by parents, who may also have been negatively influenced during the lockdown. No objective measurements, such as neurocognitive function tests, actigraphy, or polysomnography, were applied. However, considering the risk of spread, these objective measurements would have been difficult and even risky to perform during the lockdown. Third, all participants were enrolled from the clinic, not from the community, and the majority of our participants are males and children. Although gender and age differences in the reaction to the pandemic have been reported, the interpretation of our results should be done with caution.

## Conclusions

The shorter lockdown period in Taiwan resulted in fewer disturbances for children and adolescents, but there were still impacts on sleep and function. They tend to sleep longer and have less daytime sleepiness, with a more delayed sleep schedule. We also observed trends of increased emotion, behavior, and inattention problems, as well as significantly increased interference in home living. Male gender and younger age can be associated factors for sleep and functional disturbances of the lockdown. The disease control remains important during the pandemic, but governments should still pay attention to children and adolescents' psychosocial wellbeing, especially when zero clearing becomes unsustainable.

## Data availability statement

The raw data supporting the conclusions of this article are available from the corresponding author upon reasonable request.

## Ethics statement

This study was approved by the Institutional Review Board of Chang Gung Memorial Hospital (CGMH #201801537A3 and 202200182B0).

## Author contributions

Y-SH and T-CY: conception and design of the work. Y-SH, T-CY, W-CC, and IT: data collection. P-YL, IT, and W-CC: data analysis. W-CC and Y-SH: article draft, manuscript editing, and critical revision of the article. Y-SH and W-CC: final approval of the version to be published. All authors contributed to the article and approved the submitted version.

## Funding

This study was partially supported by Chang Gung Memorial Hospital Research Grants (CMRPG 3J0131, 3J0132, and 3J0133) awarded to Y-SH and (CMRPG 3L0291 and 3L0292) awarded to W-CC.

## Conflict of interest

The authors declare that the research was conducted in the absence of any commercial or financial relationships that could be construed as a potential conflict of interest.

## Publisher's note

All claims expressed in this article are solely those of the authors and do not necessarily represent those of their affiliated organizations, or those of the publisher, the editors and the reviewers. Any product that may be evaluated in this article, or claim that may be made by its manufacturer, is not guaranteed or endorsed by the publisher.
